# Comprehensive analysis of substernal lead removal: experience from EV ICD Pilot, Pivotal, and Continued Access Studies

**DOI:** 10.1093/europace/euae225

**Published:** 2024-08-30

**Authors:** Venkata Sagi, Francis Murgatroyd, Lucas V A Boersma, Jaimie Manlucu, Bradley P Knight, Christophe Leclercq, Anish Amin, Ulrika Maria Birgersdotter-Green, Joseph Yat Sun Chan, Henri Roukoz, Mauro Biffi, Haris Haqqani, Russell Denman, Christopher Wiggenhorn, Thomas R Holmes, Thomas Lulic, Paul Friedman, Ian Crozier

**Affiliations:** Baptist Heart Specialists, 836 Prudential Drive, Suite 1700, Jacksonville, FL 32207 USA; King’s College Hospital, London, UK; Cardiology Department, St. Antonius Hospital Nieuwegein, Nieuwegein, The Netherlands; Cardiology Department, Amsterdam University Medical Center, Amsterdam, The Netherlands; Division of Cardiology, London Health Sciences Centre, London, ON, Canada; Division of Cardiology, Northwestern University, Chicago, IL, USA; Cardiology Department, CHU de Rennes—Hôpital Pontchaillou, Rennes, France; Riverside Methodist Hospital, Columbus, OH, USA; Division of Cardiovascular Medicine, University of California San Diego (UCSD), San Diego, CA, USA; Prince of Wales Hospital, Chinese University of Hong Kong, Hong Kong, P. R. China; Cardiology Division, Electrophysiology Section, University of Minnesota, Minneapolis, MN, USA; IRCCS Azienda Ospedaliero-Universitaria di Bologna, Bologna, Italy; St. Vincent’s Private Hospital Northside, Chermside, QLD, Australia; St. Vincent’s Private Hospital Northside, Chermside, QLD, Australia; Cardiac Rhythm Management, Medtronic Inc., Mounds View, MN, USA; Cardiac Rhythm Management, Medtronic Inc., Mounds View, MN, USA; Cardiac Rhythm Management, Medtronic Inc., Mounds View, MN, USA; Department of Cardiovascular Medicine, Mayo Clinic, Rochester, MN, USA; Christchurch Hospital, Christchurch, New Zealand

**Keywords:** Extravascular ICD, Substernal, Lead removal, Extraction, Explant

## Abstract

**Aims:**

The extravascular implantable cardioverter-defibrillator (EV ICD) has been shown to be safe and effective for patients at risk of sudden cardiac death, but little is known about EV ICD lead removal in humans. This analysis aimed to characterize the EV ICD lead removal experience thus far.

**Methods and results:**

This was a retrospective analysis of lead removals from the EV ICD Pilot, Pivotal, and Continued Access Studies. Patients with a successful EV ICD implant who underwent lead removal were included. The main objective was lead removal success. Ancillary objectives included characterizing technique used, procedure complications, and reimplantation status. An EV ICD system was successfully implanted in 347 patients across the 3 studies (25.9% female; 53.4 ± 13.3 years; left ventricular ejection fraction: 39.7 ± 15.9). Of these patients, 29 (8.4%) underwent lead removal with a mean lead dwell time of 12.6 ± 14.3 months (0.2–58.4). The main reason for lead removal was lead dislodgement (*n* = 9, 31.0%). Lead removal was successful in 27/29 (93.1%) cases [100% (19/19) success rate <1 year and 80% (8/10) success rate >1 year post-implant]. Simple traction was used in 22/26 (84.6%) and extraction tools in 4/26 (15.4%) successful cases where technique was known. No complications were reported for any of the removal procedures. All 11 EV ICD reimplant attempts were successful.

**Conclusion:**

Complete removal of the EV ICD lead was successful in 93.1% of cases, and simple traction was sufficient in most instances. Based on these results, lead removal from the substernal space was safe and achievable up to 3 years post-implant.

## Graphical Abstract

**Graphical Abstract:**
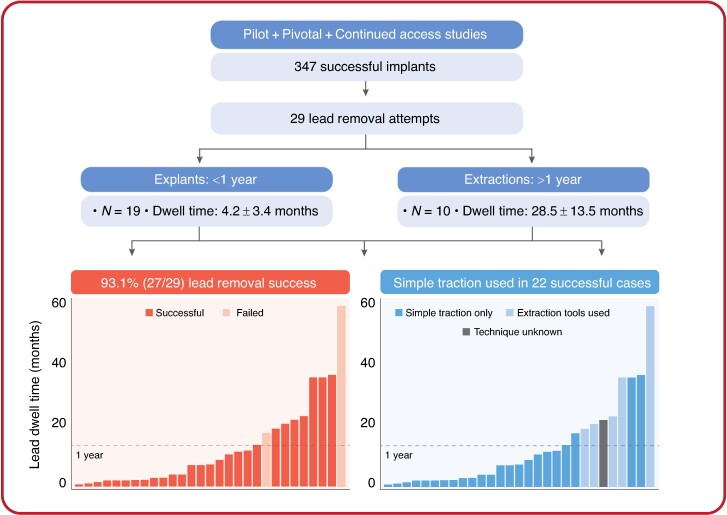
Characterizing the EV ICD lead removal experience from the Pilot, Pivotal, and Continued Access Studies. A high-level flowchart of patients included with a breakdown of the number of explants vs. extractions based on Heart Rhythm Society and European Heart Rhythm Association definitions. The two graphs summarize the overall results, including lead removal success and the technique used based on lead dwell time (*y*-axis for both graphs); each bar represents a single case and the dashed lines demarcate 1 year.

**What’s new?**

- The extravascular implantable cardioverter-defibrillator (EV ICD) has been shown to be safe and effective for patients at risk of sudden cardiac death, but data on EV ICD lead removal are lacking.
- This is the first report of EV ICD lead removal experience in humans from all of the pre-market studies.
- Lead removal was successful in 27/29 (93.1%) cases, with success in 19/19 within 1 year post-implant (explant) and 8/10 after 1 year (extractions).
- Simple traction was sufficient in most cases and in all cases within 1 year, while five with longer lead dwell times required the use of extraction sheaths due to adhesions that were located near the subxiphoid incision and diaphragmatic attachments.
- We also provide guidance and considerations for approaching EV ICD lead removal as well as lead prep techniques and sheath compatibility, critical information for physicians in the field for attempting EV ICD lead removal.

## Introduction

Implantable cardioverter-defibrillators (ICDs) are standard of care for indicated patients at risk of experiencing life-threatening ventricular arrhythmias.^1^ Transvenous (TV) ICD systems have traditionally been the only option that can provide defibrillation and pacing therapy for these patients, but placement of leads in the vasculature can result in serious complications.^2,3^ This is especially true for TV lead extraction, which can be a high-risk procedure, particularly when extracting infected or chronically implanted leads.^4^ The extravascular ICD (EV ICD) was developed as an alternative to TV ICDs, by placing the lead in the substernal space to address the concerns associated with placing leads in the vasculature.^5,6^

The EV ICD Pivotal Study established the EV ICD system as a safe and effective therapy for treating ventricular arrhythmias.^6^ However, given the unique location and novelty of the system, evidence and experience with lead removal are lacking. A recent study in sheep showed that the EV ICD lead could be removed successfully up to 3 years post-implant, with simple traction being sufficient in several cases.^7^ But more data and guidance are needed on EV ICD lead removals in humans. The current analysis was designed to report on the early experience of lead removal from the EV ICD Pilot, Pivotal, and Continued Access Studies. Patients from all three studies who underwent a lead removal attempt were combined into one cohort, with the primary endpoint being lead removal success. Additionally, we report details on procedure technique and tools used, and provide guidance and lessons learned from EV ICD lead removal experience thus far.

## Methods

### Study design and patient selection

The current study was a retrospective, observational analysis of patients from the EV ICD Pilot, Pivotal, and Continued Access Studies, who underwent an EV ICD lead removal attempt with or without generator removal.^5,6,8^ The studies were approved by the Ethics Committees at each participating institution and were conducted in accordance with the Declaration of Helsinki. All patients had either a class I or IIa indication for an ICD, provided appropriate consent before participation in the studies, and were successfully implanted with an EV ICD system. Patients who had the EV ICD lead or system removed prior to discharge due to an unsuccessful procedure or failed post-implant defibrillation testing were not included in the analysis.

### Definitions

Definitions for explant and extraction were based on the Heart Rhythm Society and European Heart Rhythm Association Guidelines.^9,10^ Lead explants were defined as removal procedures that occurred <1 year post-implant and did not require tools, whereas extractions were defined as cases where the lead was removed >1 year post-implant or required the use of tools. A successful lead removal was defined as removal of the entire lead without complication, whereas a failed lead removal included cases where a >4 cm portion of the lead was abandoned. If a <4 cm portion of the lead was left behind with no resulting complications, the removal was considered a clinical success. However, if a <4 cm portion of the lead was left behind during a removal due to infection, the case was considered a failure.

### Objectives

The main objective of the analysis was the rate of complete procedural success associated with EV ICD lead removal. Other objectives included rate of successful lead extraction or explant, characterization of tools used, reimplant status, and major and minor complications. All outcomes and complications were independently adjudicated in the clinical studies by an external data review committee, the criteria for which were consistent among studies.

### Extravascular implantable cardioverter-defibrillator lead removal procedure

Recommendations for EV ICD lead removal were not yet established for most of the removals in this analysis, given that experience with EV ICD removal is still early. Some guidance, based on physician experience, is provided in the discussion and, for use of sheaths, in the Supplementary material. To briefly describe the lead removal procedure that was generally used, the device pocket was first opened, device removed, and lead disconnected and pulled through the opened subxiphoid incision. To begin removing the substernal portion of the lead, the anchoring sutures were released. In some cases, diaphragmatic adhesions were broken up using blunt finger dissection. Next, while maintaining constant fluoroscopy of the heart, gentle and steady traction was applied to the substernal portion of the lead to remove it. If simple traction was unsuccessful, mechanical tools were used at the operator’s discretion (see Supplementary material online, *Supplemental Methods*, for more details on the use of sheaths; Supplementary material online, *Figure S1* and *Table S1*).

### Statistical analysis

Descriptive statistics were used to summarize baseline patient characteristics and endpoints. All analyses were completed using SAS software, version 9.4 (SAS Institute, Cary, NC).

## Results

### Patient characteristics

**Figure 1:**
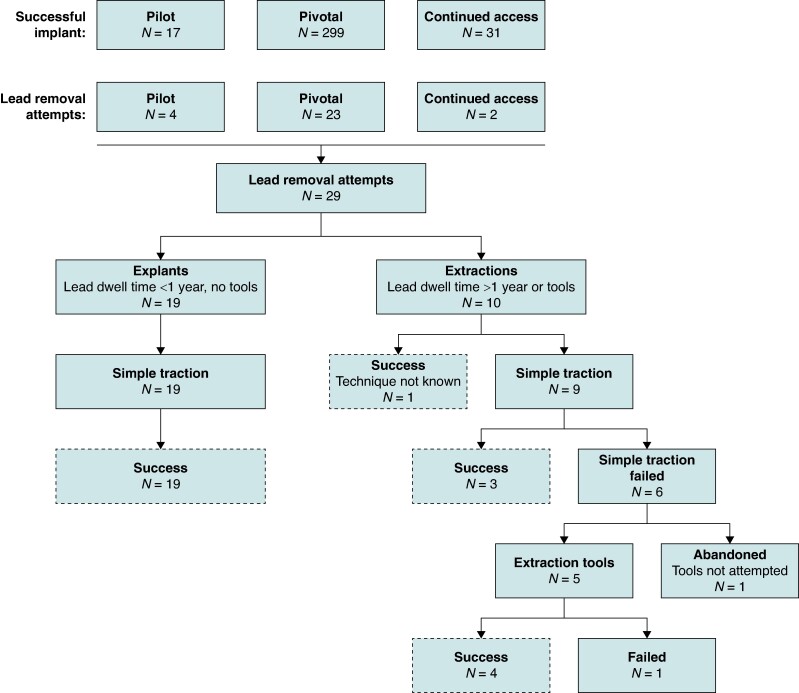
Flowchart for patients included in the analysis and outcomes. Overview of patients from the EV ICD Pilot, Pivotal, and Continued Access Studies who underwent an EV ICD lead removal procedure and general outcomes and techniques used for those procedures.

An EV ICD system was successfully implanted in 347 patients across all three studies (*Figure 1*). Of the 347 patients who underwent successful implantation of an EV ICD, lead removal was attempted in 29 (8.4%) patients (27.6% female; 54.4 ± 12.2 years old). Compared to the full cohort, the lead removal cohort had a higher rate of previous stroke, more patients with a secondary prevention indication, and a higher mean left ventricular ejection fraction (*Table 1*).

**Table 1:** Baseline patient characteristics for full and lead removal cohorts

	Successful EV ICD implant (*N* = 347)	EV ICD lead removal attempt (*N* = 29)
Mean age ± SD (years)	53.4 ± 13.3	54.4 ± 12.2
Gender (*N*, %)		
Female	90 (25.9)	8 (27.6)
ICD indication (*N*, %)		
Primary prevention	276 (79.5)	21 (72.4)
Secondary prevention	69 (19.9)	8 (27.6)
Unclassified	2 (0.6)	0 (0.0)
NYHA class (*N*, %)		w
I	83 (23.9)	6 (20.7)
II	195 (56.2)	13 (44.8)
III	25 (7.2)	3 (10.3)
IV	0 (0.0)	0 (0.0)
Subject does not have heart failure/not available	44 (12.7)	7 (24.1)
BMI mean ± SD (kg/m^2^)	28.4 ± 5.8	29.7 ± 5.0
Mean LVEF ± SD (%)	39.7 ± 15.9	46.4 ± 18.4
Cardiomyopathy^a^ (*N*, %)	287 (82.7)	22 (75.9)
Ischaemic	131 (37.8)	9 (31.0)
Non-ischaemic	115 (33.1)	7 (24.1)
Hypertrophic	46 (13.3)	8 (27.6)
Coronary artery disease (*N*, %)	159 (45.8)	13 (44.8)
Hypertension (*N*, %)	172 (49.6)	16 (55.2)
Stroke and stroke-related events^a^ (*N*, %)	26 (7.5)	5 (17.2)
Stroke, ischaemic	14 (4.0)	3 (10.3)
Stroke, haemorrhagic	1 (0.3)	0 (0.0)
Stroke, unknown origin	1 (0.3)	1 (3.4)
Thromboembolism	6 (1.7)	1 (3.4)
Transient ischaemic attack	10 (2.9)	2 (6.9)
Diabetes (*N*, %)	72 (20.7)	8 (27.6)
Renal dysfunction (*N*, %)	34 (9.8)	3 (10.3)

BMI, body mass index; EV ICD, extravascular implantable cardioverter-defibrillator; LVEF, left ventricular ejection fraction; SD, standard deviation.

^a^Events within these categories are not mutually exclusive.

The mean lead dwell time before removal was 12.6 ± 14.3 months. The main reasons for lead removal included lead dislodgement (*n* = 9), pocket infection (*n* = 5), lead fracture (*n* = 3), scheduled sternotomy (*n* = 3), pacing indication (*n* = 2), and elevated defibrillation threshold (*n* = 2) (full list of reasons provided in *Table 2*). All infections related to the EV ICD system and/or procedure resolved with standard treatment after removal of the device.

**Table 2:** Lead removal details

	All removal attempts (*N* = 29)	Explants (*N* = 19)	Extractions (*N* = 10)
Reason for lead removal, *N*			
Lead dislodgement	9	9	0
Pocket infection	5	4	1
Lead fracture	3	1	2
Scheduled sternotomy^a^	3	1	2
Pacing indication	2	2	0
Elevated defibrillation threshold^b^	2	2	0
HV impedance out of range	1	0	1
Subxiphoid incision infection	1	0	1
Oversensing/inappropriate shock	1	0	1
Aortic endocarditis^c^	1	0	1
System upgrade	1	0	1
Lead dwell time (months)			
Mean ± SD	12.6 ± 14.3	4.2 ± 3.4	28.5 ± 13.5
Lead removal success, *N* (%)			
Complete procedural success	27 (93.1)	19 (100)	8 (80)
Failed procedure	2 (6.9)	0	2 (20)
Extraction technique, *N*			
Simple traction only	23	19	4
Byrd telescoping dilator sheath	2	0	2
Spectranetics TightRail sheath^d^	3	0	3
Philips GlideLight Laser sheath^d^	1	0	1
Not available	1	0	1
Reimplant device, *N*			
EV ICD lead or system	11	9	2
Transvenous	5	3	2
S-ICD	2	2	0
Not known	11	5	6

EV ICD, extravascular implantable cardioverter-defibrillator; HV, high voltage; SD, standard deviation; S-ICD, subcutaneous ICD.

^a^Unrelated to the EV ICD system or implant procedure; one was for septal myectomy, one for heart transplant, and one for aortic valve repair.

^b^For one pilot patient, elective DFT testing was performed due to increased HV impedance and was successful at 40 J, but physician decided to explant; second was a Continued Access patient that failed DFT testing 6 days post-discharge and the patient was reimplanted with a new lead.

^c^Unrelated to the EV ICD system or implant procedure.

^d^In one extraction case, both a TightRail sheath and GlideLight laser sheath were used.

### Lead removal success

**Figure 2:**
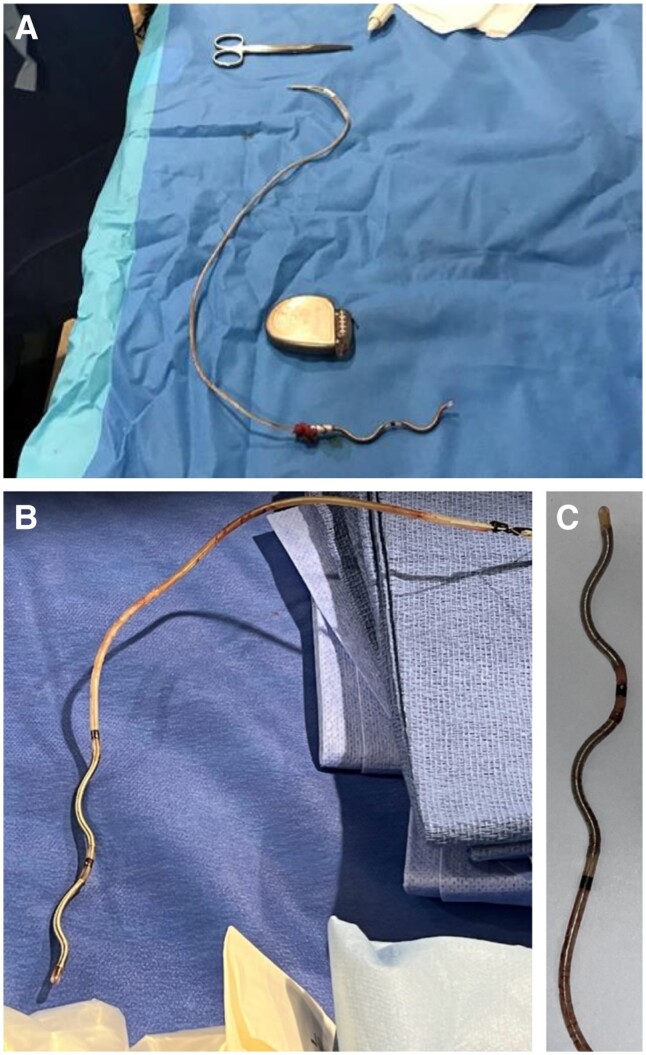
Example images of EV ICD leads after being removed. Image of a full EV ICD system that was explanted at 3.7 months post-implant (*A*). Image of full lead extracted at 24.7 months post-implant (*B*). Epsilon lead tip after extraction 35.9 months post-implant (*C*).

**Figure 3:**
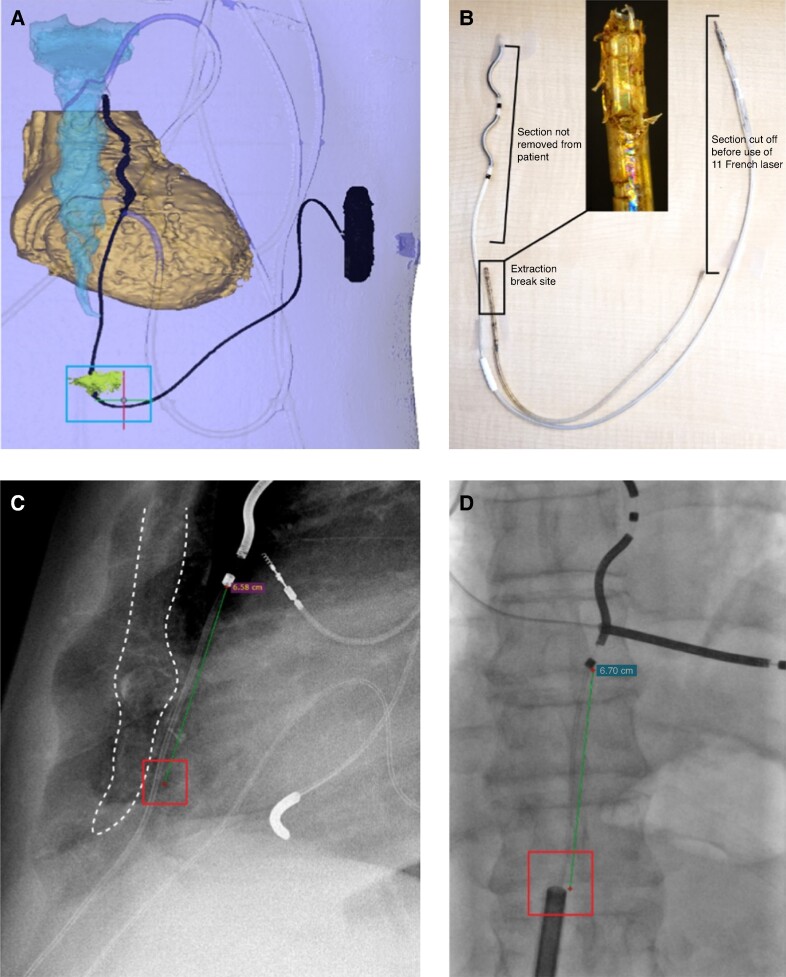
Images of chronic EV ICD lead that broke during extraction and was partially abandoned. 3D image of EV ICD lead placement before removal procedure; box at bottom of image demarcates the low midline subxiphoid incision site near the area with tough adhesions (*A*). Image of extracted portion of the lead next to a full-length lead for reference; close-up image is provided showing where extracted portion separated from abandoned portion of the lead (*B*). Lateral X-ray (*C*) and anteroposterior fluoroscopy (*D*) images showing sheath advancement into site of tough adhesions (boxes); sternum outlined with a dashed line in (*C*).

The EV ICD lead removal success rate was 93.1% (27/29), with a 100% (19/19) and 80% (8/10) success rate for explants and extractions, respectively (*Table 2* and *Figure 2*). In one failed extraction at 16.3 months post-implant, the patient required an upgrade to a cardiac resynchronization therapy defibrillator. The lead removal attempt was not performed at the original study site, and the full lead was left in place after removing the generator and only attempting simple traction of the lead. In the second failed extraction case at 58.4 months post-implant, it was determined, in retrospect, that more of an attempt could have been made to break apart adhesions located at and below the diaphragm before proceeding with extraction. As a result, a 6.4 cm portion of the distal end of the lead was abandoned in the substernal space after mechanical and laser sheaths failed to advance past the adhesions (*Figure 3*; Supplementary material online, *Video S1*). The sheath interacted with the lead in a non-coaxial orientation such that lead damage occurred and eventually broke at the site of adhesions, highlighting the need to have full lateral and anteroposterior fluoroscopy viewing angles available during sheath advancement. Neither failed case was related to or in response to an infection. In both cases, abandoning part or all of the lead did not result in any adverse outcomes for the patients.

### Lead removal procedure details

Data on the lead removal technique and tools used was available for 26 of the 27 successful cases. Of the 26 successful lead removals with available data, simple traction was sufficient for complete removal in 22 (84.6%) cases including 3 extractions at 12.9, 35.9, and 36.8 months post-implant (see Supplementary material online, *Video S2*). Extraction tools were used in 4/26 (15.4%) successful cases where technique was known and included a Spectranetics TightRail sheath (*n* = 2; 21.9 and 24.7 months) and a Byrd dilator sheath (*n* = 2; 18.8 and 35.9 months) (see Supplementary material online, *Video S3*). For the three removals due to a scheduled sternotomy, the lead was removed by the cardiac surgeon at time of sternotomy in two cases, one by simple traction and the other where removal technique was not known. As discussed previously for the two failed cases, only simple traction was attempted in one failed case, and in the second failed case, both the Spectranetics TightRail sheath and Philips GlideLight Laser sheath were used. Additional incisions were not required for any of the removals.

### Device replacement

**Figure 4:**
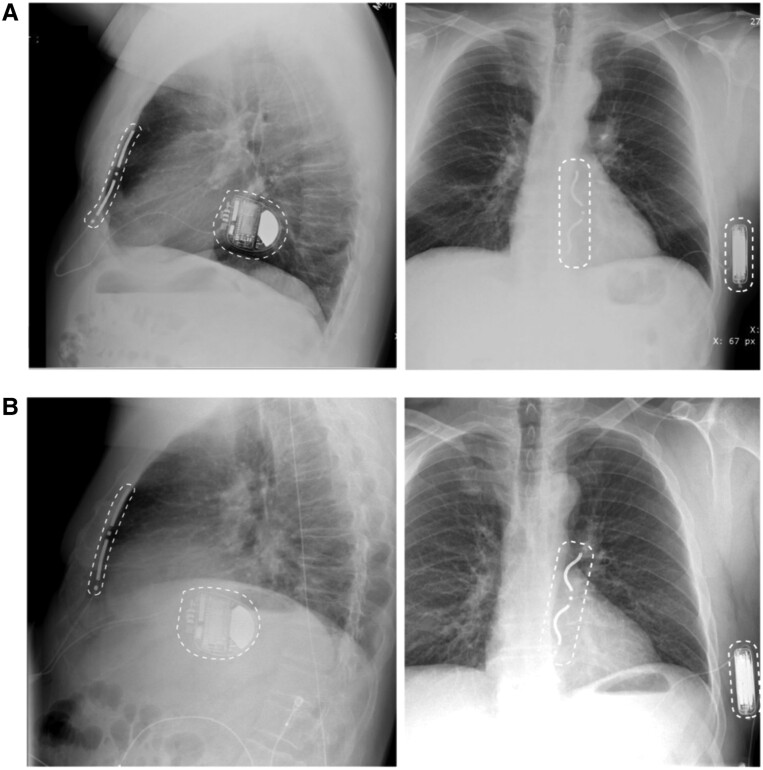
X-ray imaging of original EV ICD implant before extraction and replacement EV ICD system. Lateral and frontal X-rays of the original EV ICD implant before extraction (*A*). Lateral and frontal X-rays of replacement EV ICD that was implanted after extraction 35.9 months post-implant (*B*). The can and epsilon lead tip are indicated with a dashed outline.

Eighteen (62.1%) patients underwent successful reimplantation of a new lead or system after removal. A new EV ICD lead was reimplanted in 11 (37.9%) patients, 2 (6.9%) of whom also received a replacement generator (*Figure 4*). This was successful in all cases: the substernal tunnelling procedure encountered no adhesions or resistance, electrical parameters were stable, and all subjects underwent successful defibrillation threshold testing. The new leads have remained stable to date. Five further patients received TV systems, and two received a subcutaneous ICD (S-ICD). The remaining 11 patients exited the study after lead removal and so reimplant status is not known for these patients.

### Complications

No major or minor complications occurred during any of the lead removal procedures. One incision site haemorrhage was observed, which resolved after applying pressure with sterile gauze.

## Discussion

This is the first report on EV ICD lead removal in humans and the first to provide recommendations on approaching lead removal from the substernal space. We show that complete lead removal was achieved in 93.1% (27/29) of patients up to 3 years post-implant, and simple traction was successful in 78.6% (22/28) of cases where technique data were available, including 3 extractions ranging from 1 to 3 years post-implant (*Graphical Abstract*). All 19 lead removals performed <1 year post-implant were successful while 8 of 10 were successful past 1 year. Extraction tools were used in half of extractions (>1 year dwell time), indicating the development of adhesions as dwell time increases. Complete lead removal was unsuccessful in two cases. Importantly, no complications, including cardiac or vascular injury, occurred as a result of any of the EV ICD lead removal attempts.

### Learnings from failed cases

In both failed extractions, the recommendations for EV ICD lead removal outlined in this report were not followed or not available. In one failed extraction, the procedure was not performed at a study site or by an operator familiar with using sheaths in the substernal space and the lead was abandoned after only attempting simple traction. This case highlights our recommendation that the operator be comfortable with, or have baseline knowledge, implanting the EV ICD lead or operating in the substernal space before attempting lead removal. In the second failed case, adhesions were only partially dissected, and a distal portion of the lead broke off due to sheath–lead interactions and loss of coaxial alignment at the site of tough adhesions. This case illustrates two important recommendations for EV ICD lead removal. First, any adhesions, which are usually concentrated around the distal portion of the lead body, proximal to Ring 2, near the diaphragmatic attachments and the subxiphoid incision, should be broken up as much as possible with finger dissection before attempting lead removal. It is worth mentioning that no notable adhesion formation within the substernal space has been observed in EV ICD lead removal cases thus far. Secondly, full anteroposterior and lateral fluoroscopic views are necessary to appreciate the coaxial alignment of the sheath with the lead body especially when transitioning from subcutaneous to substernal tissue planes and when navigating through adhesions. These recommendations should be followed in all cases but are especially critical in cases with long dwell times where more adhesions are likely to have formed. High-volume lead extraction centres may be best positioned for removing a chronically implanted lead with adhesions, so long as they also have appropriate EV ICD training. In addition to these recommendations, it is worth noting that the EV ICD lead has a different construction than a TV lead and preparation for use of extraction tools should reflect this to obtain optimal rail strength. More information on lead prep and sheath compatibility can be found in the Supplementary material.

### Extravascular implantable cardioverter-defibrillator lead removal in context

Up to this point, the only published study on EV ICD lead removal was a 3-year extraction experience in sheep that compared outcomes to TV lead extraction.^7^ In that study, all 19 EV ICD leads were extracted successfully (dwell times ranging from 1 to 3 years) with 3 (15.8%) leads being removed with simple traction and the other 16 (84.2%) requiring the use of tools, while all TV lead extractions required the use of extraction tools. No additional incisions were needed for extraction of the EV ICD leads in the sheep study. Similar results are shown in the human experience reported herein, where 3 of 10 extractions (>1 year) were successful with simple traction and no additional incisions were needed in any case.

While comparing EV ICD to TV lead removal is important, the procedure is more comparable to removing an S-ICD lead, given that both are extravascular.^11^ Also, both the EV ICD and S-ICD have a lumenless design, which increases the tensile strength for lead removal.^12^ There have been several reports on S-ICD lead removal. In a recent multicentre study in France, 31 of 32 (96.9%) S-ICD lead removal attempts were successful, 19 (59.4%) of which were achieved with simple traction.^13^ Both numbers are in line with what we show for EV ICD with a 93.1% success rate and 78.6% (where technique was known) being successful with simple traction. An Italian centre reported a 100% success rate in 71 S-ICD lead removal cases where simple traction was used in 84% of cases.^14^ Assessing lead removal technique for S-ICD lead removal, a systematic review showed, in cases with available data, simple traction was used in 79.8% of these cases, 11.3% used tools, and 8.9% required an additional incision.^15^ The tools reported in the review were consistent with those used in our current report on EV ICD (i.e. TightRail). Similarly to EV ICD, there were no reported complications with lead removal across any of the S-ICD studies. Contrary to EV ICD lead removal experience, 11/124 (8.9%) S-ICD lead removals with known technique required a third incision compared to none for EV ICD. While what has been reported for S-ICD lead removal is comparable to what we report, the experience with EV ICD is still early relative to S-ICD, and more data with dwell times >3 years are needed.

Patients that could derive maximal benefit from extravascular lead placement are the same as those at high risk for TV lead removal. Young patients have a higher chance of experiencing complications from TV lead extraction due in part to having longer implant durations.^16^ End-stage renal disease patients need their vascular space preserved for the purpose of dialysis, and lead removal is especially complicated in this population due to increased calcium deposition on the TV lead. Active patients, regardless of age, may be more likely to sustain lead fractures requiring the need for lead revisions and removal.^17^ Thus, the EV ICD system may be a desirable alternative to TV ICDs for patient groups such as these that are at a higher risk of experiencing serious complication during lead removal. The benefits of using an extravascular system should also be considered when determining whether to reimplant with an EV ICD lead or system after removal. Removal and reimplantation of the EV ICD lead or system can be performed concomitantly unless the reason for removal is infection, in which case reimplantation should be deferred until after the resolution of the infection.

### Limitations

Most of the lead removal attempts were performed within the confines of a clinical study at high-volume implant centres, some of which may also be high-volume extraction centres; this is a possible limitation of these results given high-volume extraction centres have shown a higher lead removal success rate.^18^ The results are retrospective with a small sample size. Additionally, the number of chronic extractions is small. A larger sample size of longer-term extractions is needed to confirm the safety of chronic EV ICD lead extraction. Given that experience with EV ICD is still early, more data from leads with a dwell time >3 years, a predictor of high risk for TV ICD lead extraction, are needed. Also, not all procedure information was available for each case such as procedure time, fluoroscopy time, specific tools used in one case, and whether a surgeon was present; these would help to assess how EV ICD lead removal compares to other systems in future studies.

## Conclusions

Complete EV ICD lead removal was successful in 93.1% (19/19 explants, 8/10 extractions) of patients using simple traction in the majority of cases, and no complications, including cardiac injury, occurred because of any of the removal attempts. Additionally, retunnelling to reimplant with a new EV ICD lead after removal was successful in all 11 attempts without complication. These findings demonstrate that removal of the EV ICD lead was safe and achievable, but more experience with chronic leads implanted for longer than 3 years is needed.

## Supplementary Material

euae225_Supplementary_Data

## Data Availability

The data underlying this article cannot be shared publicly in order to maintain the privacy of individuals who participated in the study. For any special request, please contact the corresponding author.
