# Supplementary material for: Comprehensive analysis of substernal lead removal: experience from EV ICD Pilot, Pivotal, and Continued Access Studies
Source: Europace. 2024 Aug 30;26(9):euae225. doi: 10.1093/europace/euae225 (PMC11420630; doi:10.1093/europace/euae225)
Supplement: euae225_Supplementary_Data [file euae225_supplementary_data.zip › Supplemental Figures & table.docx]

**Supplemental Figures**

**
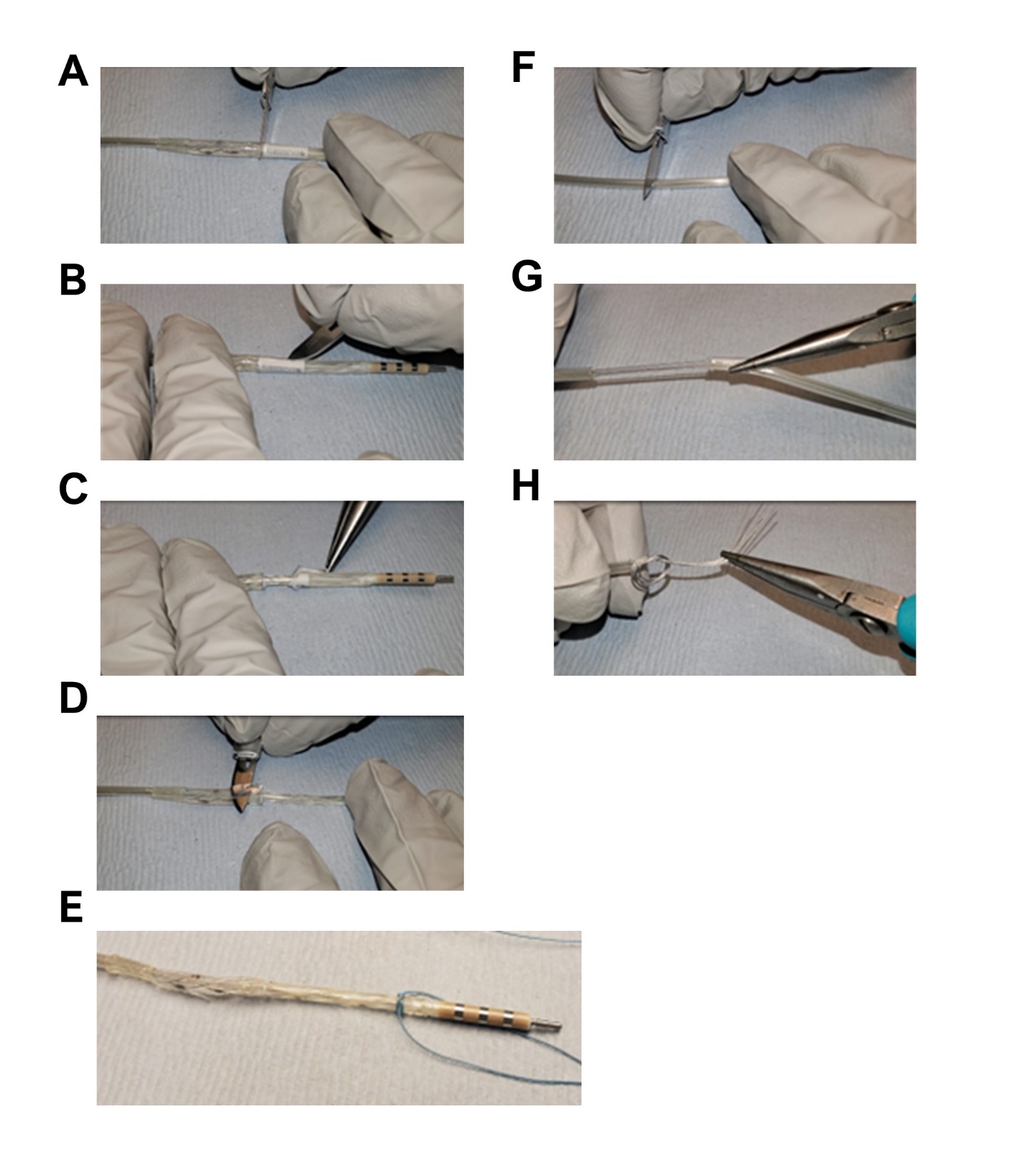
**

**Supplemental Videos**

**(double click the mp4 link to view videos)**

**Supplemental Video 1.** Fluoroscopy imaging from failed extraction 4.8 years post-implant showing inability of sheath advancement past tough adhesions. Tight rail is seen at bottom of the frame as dark object.

**Supplemental Video 2.** Fluoroscopy imaging from successful extraction 3 years post-implant using simple traction only.

**Supplemental Video 3.** Fluoroscopy imaging from successful extraction 3 years post-implant using Byrd telescoping dilator sheath.

**Supplemental References**

**1.** Vatterott PJ, Mondesert B, Marshall M, Lulic T, Wilkoff BL. Mechanics of lumenless pacing lead strength during extraction procedures based on laboratory bench testing. Heart Rhythm 2023;20:902-909.

**Supplemental Tables**

**Supplemental Table 1: EV ICD lead prep method and sheath compatibility**

| **Prep technique** | **Max OD** | **Rail Strength (lbs)** | **Compatible sheaths** | **Some friction** | **Incompatible sheaths** |
| --- | --- | --- | --- | --- | --- |
| Connector retained | 11 - 12 Fr | 15 - 20 | - TightRail 13 Fr - Evolution RL 13 Fr | - TightRail 11 Fr - Evolution RL 11 Fr - GlideLight 16 Fr | - GlideLight 14 Fr & 12 Fr - TightRail 9 Fr - Evolution RL 9 Fr |
| Connector removed | 9 - 10 Fr | 10 - 20 | - TightRail 13 Fr - Evolution RL 13 Fr - GlideLight 16 Fr - TightRail 11 Fr - Evolution RL 11 Fr | - GlideLight 14 Fr - TightRail 9 Fr | - GlideLight 12 Fr - Evolution RL 9 Fr |
| *Not all sheaths designs and diameters have been studied. This table represents dimensional compatibility for the suggested prep techniques and does not imply clinical success.  Fr: French; OD: outside diameter | | | | | |

**Supplemental Figures andFigure Legends**

**Supplemental Figure 1. EV ICD lead preparation for use of extraction sheaths.**

Images depicting lead preparation steps for both prep techniques: panels A-D for option 1 (retaining the terminal pin) and panels F-H for option 2 (removing terminal pin). Panel E depicts constrictor knot suture tied to a lead that was prepped using option 1.
